# Assessment of Cognitive Reserve using Near Infrared Spectroscopy

**Published:** 2022-05-19

**Authors:** Andrei V. Medvedev

**Affiliations:** Center for Functional and Molecular Imaging, Department of Neurology, Georgetown University Medical Center, Washington DC, USA

**Keywords:** fNIRS, Near infrared spectroscopy, Cognitive reserve, Prefrontal cortex, Functional connectivity, Resting state

## Abstract

Cognitive reserve (CR) is the ability to preserve cognitive functions in the presence of brain pathology. In the context of Alzheimer’s disease (AD), patients with higher CR show better cognitive performance relative to brain damage therefore higher CR reduces the risk of dementia. There is a strong need to develop a neurophysiological biomarker of CR given the growing interest in understanding protective brain mechanisms in AD. FMRI studies indicate that frontoparietal network plays an important role in cognitive reserve. We calculated intraregional functional connectivity of lateral prefrontal cortex (FC LPFC) using functional near infrared spectroscopy (fNIRS) in the resting state of 13 healthy individuals who were also assessed for IQ and motoric skills (the Purdue Pegboard test, PPT). FC LPFC was found to positively correlate with IQ (a proxy measure of cognitive reserve) while showing a lack of or negative correlation with the PPT scores. The results demonstrate that the cost-effective, noninvasive and widely applicable fNIRS technology can be used to evaluate cognitive reserve in individuals at risk for and patients with AD with possible numerous applications in the context of healthy aging and other age-related cognitive disorders.

## 1. Introduction

Cognitive processes and their changes in healthy and pathological aging have been investigated in detail over the past few decades which led to the development of new theoretical models of aging. Existing data show a complex picture of age-related changes in cognitive functions, which can include both the relative stability and deterioration. Thus, many perceptual and cognitive processes demonstrate a general slowing e.g., leading to difficulties in finding the right word during speech production. Age- and pathology-related microstructural changes in gray and white matter [1–3] may underlie the observed reduction in processing speed, working memory capacity and hearing loss which reduce cognitive abilities [4–6]. Nevertheless, despite those deficits, older people may show a relative stability of certain cognitive functions despite the observed microstructural pathology. This suggests the existence of both dysfunction and possibly compensation in functional networks [7–9] and naturally raises the question of substitute or compensatory mechanisms by which this stability, commonly referred to as cognitive reserve (CR), is achieved. The concept of CR was introduced by Stern to explain the absence of a direct relationship between the degree of brain pathology or damage and the clinical manifestation of that damage [9]. For example, in several studies it has been demonstrated that up to 25% of senior people have unimpaired cognitive functioning revealed by neuropsychological testing prior to death while meeting full pathologic criteria for AD (for review, see Stern, 2009). This suggests that this degree of pathology does not necessarily lead to clinical dementia. The concept of cognitive reserve posits that individual differences in functioning of neural networks allow some people to withstand the pathological process and maintain a relatively high level of cognitive functions. The CR concept is supported by numerous epidemiological studies. For example, Valenzuela and Sachdev in their meta-analysis reviewed 22 papers reporting cohort studies of the effects of education, occupation, premorbid IQ and mental activities in incident dementia [10]. Ten out of 15 studies revealed a significant protective effect of education; 2 out of 2 showed a protective effect of premorbid IQ; 9 out of 12 demonstrated a protective effect of occupational attainment and 6 out of 6 showed a protective effect of engaging in leisure activities (reviewed in [9]). The authors concluded that higher CR (as measured by IQ and years of education) was significantly correlated with a lower risk for incident dementia. Different neural mechanisms may underlie cognitive reserve and Stern (2009) suggested two major components, neural reserve reflecting just individual differences in neural networks and neural compensation which requires alterations in cognitive processing in order to cope with brain pathology. Thus, a more efficient use of brain networks and/or recruitment of alternate brain networks may lead to the development of CR. Numerous studies indicate that environmental enrichment and specific life experiences such as educational activities and occupation play an important role in developing CR and reducing risk of dementia [9].

### 1.1 Measures of cognitive reserve

To assess CR, various measures of socioeconomic status are used such as occupational, educational (including degree of literacy) and leisure activity. Many studies suggest that IQ is a powerful measure of reserve because it reflects innate intelligence [11].

Evidence is emerging that functional architecture of the brain is relatively stable in a variety of cognitive tasks and the resting state. Recent brain imaging studies have demonstrated the existence of several networks within the brain which can be distinguished by correlated activity of their nodes over time within each network and uncorrelated or anti-correlated activity between networks. Those networks have been suggested to represent an essential feature of the functional architecture of human brain [12–14]. Thus, their analysis is important for our understanding of normal and impaired cognitive functions. This network organization seems to exist independently of the functional state of the brain because the same patterns of correlated and anti-correlated activity are observed during cognitive task performance as well as in the absence of any task or stimulus i.e., in the resting state. Slow (<0.1 Hz) fluctuations of brain activity correlated within the network can be recorded by measuring BOLD fMRI signal [15]. Similar signals can be detected in blood hemoglobin levels when measured by functional near-infrared spectroscopy (fNIRS) [16–30]. Networks of brain regions that are activated during cognitive tasks (e.g., working memory) have been labeled as “task-positive” (TPNs). Other networks, by contrast, showing deactivation during cognitive tasks but activation in the resting state have been labeled as “task-negative” (TNNs or the “default mode network”, DMN). The TPNs and TNNs show either no correlation or even anti-correlative relationship to each other that is observed during both task performance and the resting state [12].

The strength of functional interactions within and between networks (network connectivity) has been suggested to be one of the key determinants of cognitive abilities in general and more specifically, cognitive control which is the ability to effectively control thoughts and behavior in everyday life. Cognitive control can be measured by working memory capacity and general fluid intelligence [31]. The general feature of cognitive control is its limited capacity which predicts important life relevant outcomes such as academic and professional success. The fundamental property of cognitive control is its extraordinary ability to adaptively organize a wide variety of tasks thus providing a central mechanism allowing an individual to switch between different tasks in a highly flexible manner as well as to learn new tasks. There is an emerging consensus that there is a core set of brain structures which is centrally involved in cognitive control- a cognitive control network (CCN). This functional network consists of the lateral prefrontal cortex (LPFC) and the posterior parietal cortex (PPC), two parts of the frontoparietal network (FPN). FPN has been variously termed the cognitive control network or system [31], the superordinate cognitive control network [32], the multiple-demand system [33] and the task-positive network [12]. The cognitive control network has received attention in recent studies [34–36]. Thus, Cole et al. (2012) have demonstrated statistically significant correlations between general fluid intelligence and positive Global Brain Connectivity of the frontal part of the cognitive control network that is, the lateral prefrontal cortex, but the lack of such correlation for the parietal part of the FPN, the medial posterior parietal cortex. Franzmeier et al. (2018) have shown that higher levels of left frontal cortex connectivity are correlated with greater education and, importantly, with slower decline of memory in patients with AD. These results demonstrate that higher left frontal cortex connectivity plays a significant role in higher resilience against the development of cognitive impairment at the early stages of Alzheimer’s disease.

The goal of this study was to demonstrate that near-infrared spectroscopy can be used to measure intraregional functional connectivity of the lateral prefrontal cortex (FC LPFC) and to explore whether this connectivity correlates with cognitive and behavioral performance specifically, the IQ, a proxy measure of cognitive reserve, as well as the Purdue Pegboard Test (PPT) in healthy individuals.

## 2. Materials and Methods

**Figure 1:**
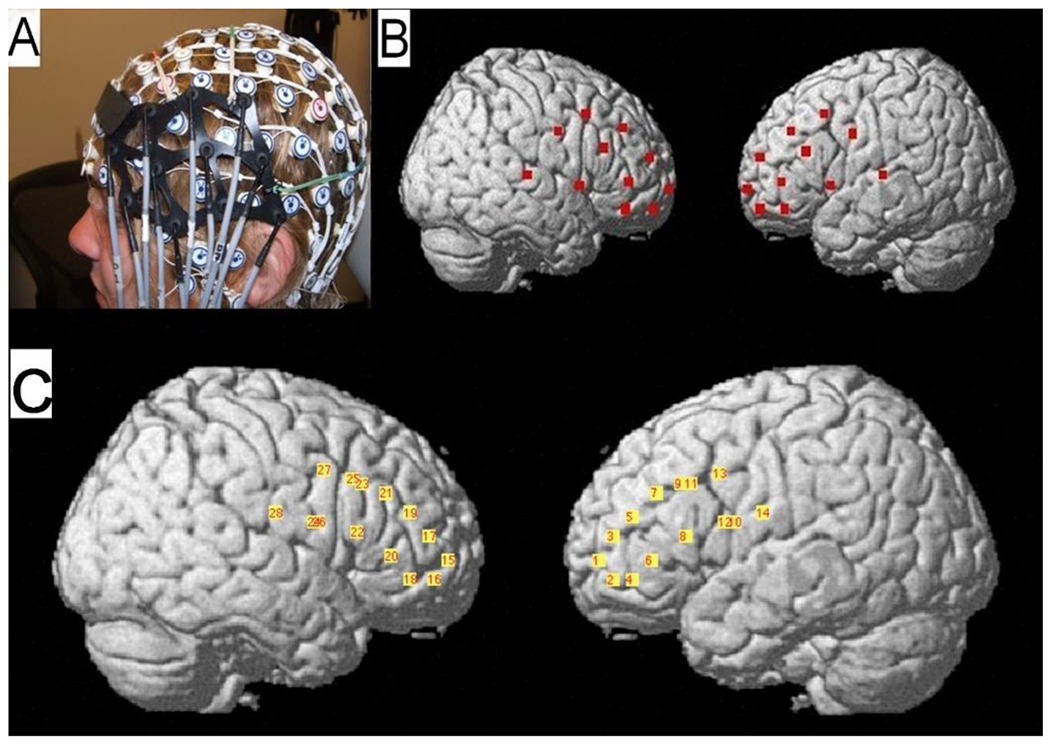
(A) Combined optical and EEG recording probe. (B) Positions of all optical fibers were digitally co-registered in reference to the standard locations of the 10-20 electrode placement system and then projected on the cortical surface using the fNIRS-SPM toolbox. (C) Positions of all analyzed optical ‘channels’ i.e., the midpoints between laser sources and the nearest detectors (located at ~3 cm from the corresponding source).

In this study, we used the resting state data from the same cohort of adult subjects which were previously analyzed in the context of hemispheric asymmetry of functional connectivity (Medvedev, 2013). The details of the methods can be found there. Briefly, 13 right-handed subjects reported as being in good health, having normal (or corrected to normal) vision and without medications and signed a consent form approved by the Georgetown University Institutional Review Board. Before experiments, they undertook a battery of cognitive and behavioral tests which included measures of IQ (Wechsler Abbreviated Scale of Intelligence), handedness and Purdue Pegboard Test which is commonly used for finger and hand dexterity and bimanual coordination. The board used in the PPT has two parallel rows of 25 holes each and subjects have to place as many pins as possible into the holes within the 30-second time interval. There are three subtests for the left hand, right hand and both hands. The 4^th^ subtest requires subjects to use both hands and construct “assemblies” consisting of pins, collars and washers. The test scores are just the numbers of pins inserted or assemblies constructed. Thus, the greater scores indicate a better manual coordination and dexterity. In the current analysis, the score for the performance with both hands (PP Both Hands) and the score for the assembly subtest (PP Assembly) were used. Subjects were scanned during 4-8 min rest (with eyes closed) before engagement in a cognitive task. The minimum duration of the rest period was 4 min but it was extended up to 8 min if visible motion artifacts were present during recording. Four-minute artifact-free (as determined by visual inspection) segments were selected for further analysis. Task-related data have been published elsewhere [27] and for the current study, the resting state data were analyzed. Optical signals were recorded using a continuous-wave fNIRS instrument CW5 (TechEn, Milford, MA) with two 14 × 8-cm probes, each accommodating 11 optodes with three dual-wavelength (690 and 830 nm) laser sources and eight detectors for each hemisphere (the source-detector separation was 3 cm). Optical probes were placed on the scalp on top of a 128-channel HydroCel EEG net (Electrical Geodesics, Inc. (EGI), Eugene, OR) with locations F3/4-F7/8-C3/4 used as reference points. The EEG net provided a consistent frame of reference for positioning optical probes. Optode coordinates in 10–20 reference space were estimated by triangulation with the three nearest EEG electrodes, using the electrode coordinates from the template provided by the EGI. The NFRI software package [37] was then used to generate interpolation kernels for projection of channel data onto the brain surface, with interpolation only taking place between channels on the same probe (Figure 1). Using information relating the 10-20 locations to the underlying cortical areas and the optode localization procedures [38], we established that source-detector pairs from the lower half of the left/right probe reflected the activity of the left/right inferior frontal gyrus (IFG) while source-detector pairs from the upper half reflected the activity of the left/right middle frontal gyms (MFG) (Figure 1). Thus, optical probes covered LPFC, the frontal part of the frontoparietal cognitive control network.

Preprocessing of optical signals included the standard steps for artifact removal using Independent Component Analysis (ICA) and bandpass filtering (0.01-0.1 Hz). As we have shown previously, the ICA can be used to effectively remove a nonspecific contribution from the superficial layers (skull and scalp; Medvedev et al., 2008). This contribution usually contains global systemic physiological artifacts such as cardiac and respiratory signals as well as noise. The narrow-band cardiac and respiratory signals as well as the broadband noise can be detected by ICA with the subsequent spectral analysis of all independent components and removal of artifactual ones [39]. Motion artifacts were removed using a novel Temporal Derivative Distribution Repair (TDDR) method based on robust regression [40]. For each participant, we calculated intraregional functional connectivity of the lateral prefrontal cortex following the procedure described in (Cole et al., 2012) adapted for our optical data [34]. Briefly, FC LPFC was determined first for each optical channel covering the left and right lateral PFC). Brain activity was analyzed separately for oxygenated hemoglobin (HbO), deoxygenated (reduced) hemoglobin (HbR) and total hemoglobin (HbT = HbO + HbR). Activity of each prefrontal channel was correlated with all other channels and all positive correlation coefficients were z-transformed (by Fisher’s transform) and averaged over all PFC channels (as per Cole et al., 2012). Only positive correlations were taken into account because positive and negative correlations may cancel each other out during averaging. Also, the underlying assumption is that it is positive correlations that indicate a functionally cooperative, rather than counteractive, interaction between brain structures. For each individual, the resulting FC LPFC numbers (separately for HbO, HbR and HbT) were then correlated with his/her IQ (WASI full score) and two scores of the two PPT subtests namely, the PP Both Hands score and the PP Assembly score.

## 3. Results

**Figure 2:**
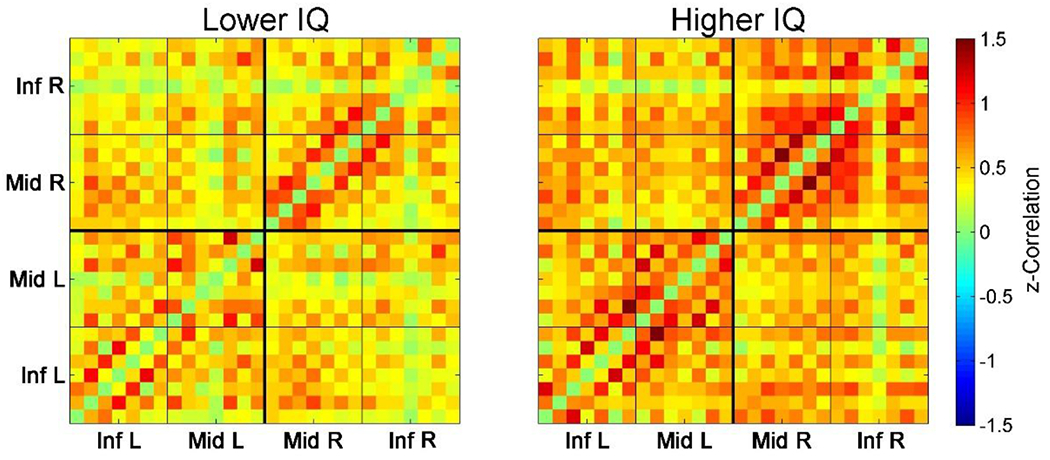
Connectivity matrices for the 1^st^ quartile (3 subjects with the lower IQ scores, *left*) and the 4^th^ quartile (3 subjects with the higher IQ scores, *right*). Each (28 x 28) matrix represents Fisher-transformed correlations calculated between all 28 optical channels pairwise. Correlation between channels v1 and v2 is represented by color at point with x-coordinate = v1 and y-coordinate = v2 (correlations for each channel to itself as well as all negative correlations are intentionally zeroed and not included into the analysis, see text for further details). Matrices are symmetrical along the main diagonal from bottom-left to top-right. Inf L and Inf R are left/right inferior frontal gyrus (IFG), Mid L and Mid R are left/right middle frontal gyrus (MFG).

First, we divided all subjects into quartile groups according to their IQ and then compared prefrontal FC LPFC values between the 1^st^ quartile with the lower IQ scores (3 subjects, mean IQ = 113 ± 4.3) and the 4^th^ quartile with the higher IQ scores (3 subjects, mean IQ = 131 ± 1.2; the difference in IQ compared to the 1^st^ quartile is significant: t = 7.0, p < 01). The HbT-based connectivity maps revealed higher correlations for the majority of pairwise connections in the group with higher IQ (Figure 2). The corresponding FC LPFC values for these two groups were 0.36 ± 0.16 (the lower IQ group) and 0.69 ± 0.18 (the higher IQ group). The difference in FC LPFC between groups was significant (t = 2.3; p < 0.05).

**Figure 3:**
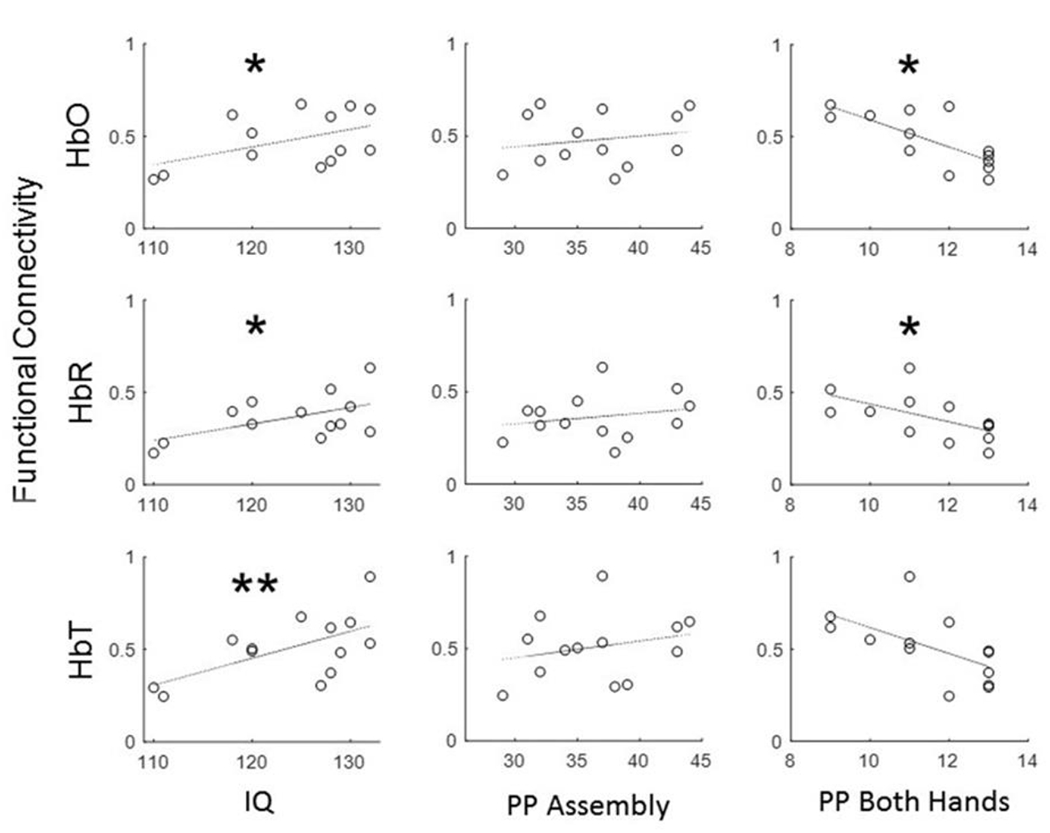
Relationship between intraregional functional connectivity of the prefrontal cortex (FC LPFC) and the results of cognitive and behavioral tests (WASI and Purdue Pegboard Test) across a cohort of 13 healthy adult individuals. FC LPFC showed positive correlation with IQ for all three optical signals and the lack of or negative correlation with motoric skills as measured by Purdue Pegboard Test. HbO, HbR and HbT are oxygenated, deoxygenated and total hemoglobin, respectively. PP Assembly and PP Both Hands are scores from the corresponding subtests of the Purdue Pegboard Test. Significance of linear regressions: one asterisk, p < 0.05; double asterisk, p < 0.01, FDR-corrected for multiple comparisons with 3 dependent variables (IQ, PP Assembly and PP Both Hands) and 3 tests for each of the three hemoglobin contrasts).

We then correlated individual FC LPFC values with the corresponding IQ and PPT scores across all subjects. Prefrontal functional connectivity (for all three hemodynamic signals) was found to positively correlate with the individual IQ scores with the highest significance for the total hemoglobin (Figure 3). Interestingly, the FC LPFC values did not correlate with the PP Assembly score and showed a significant negative correlation with the PP Both Hands score for HbO and HbR (Figure 3).

## 4. Discussion

The use of functional near infrared spectroscopy in the studies of brain networks is growing in popularity. One limitation of fNIRS is that it cannot reach deep brain structures. However, all global brain networks have large cortical components [41]. This is especially true for the cognitive control network the major component of which is the lateral prefrontal cortex easily accessible by fNIRS. In the previous fNIRS study, we have shown that activation in PFC [38] scales linearly with working memory load thus confirming the adequacy of optical imaging for functional assessment of the frontoparietal network. The advantage of fNIRS is that functional connectivity can be measured through temporal correlation between two raw time series with minimal preprocessing because optical signals are much less sensitive to motion (a significant problem in fMRI studies) and have higher sampling rate (50 Hz with the CW6 instrument) than fMRI (0.3-0.5 Hz). This prevents aliasing of higher frequency activity such as respiratory (~0.2 Hz) and cardiovascular (~1 Hz) signals into low frequency (<0.1 Hz) fluctuations through which functional connectivity is usually assessed. Higher temporal resolution of fNIRS and its ability to measure relative changes in concentrations of two forms of hemoglobin separately (oxygenated, HbO and deoxygenated or reduced, HbR; their sum also determines the relative changes in tissue total hemoglobin) provide more information about brain hemodynamics. Along with the portability of fNIRS allowing its use even in neonates at bedside and senior populations, the existing data demonstrate the capability of fNIRS to provide effective tools complementary to the gold standard BOLD fMRI, to study the architecture of brain functional networks.

As Cole et al. (2013) emphasize, the most intriguing and mysterious property of the frontoparietal network is its ability to meaningfully contribute to a wide variety of task demands and moreover, being most active during the implementation of novel and non-routine tasks that the system has not had a chance to adapt to by practice or during evolution [31]. These authors further demonstrated that such an extraordinary ability of the FPN results from a specific anatomical and functional organization of this system which involves *flexible hubs* i.e., brain regions that can flexibly and rapidly change their patterns of functional connectivity with other brain networks in order to achieve cognitive control across a variety of tasks (the flexible hub theory). The earlier study by the same group has shown that global connectivity of prefrontal cortex predicts cognitive control and intelligence. Recent studies by Franzmeier et al. (2017; 2018) addressed the role of cognitive control network in the development and maintenance of cognitive reserve at the early stages of AD. Using fMRI these authors demonstrate that resting-state global functional connectivity of FPN correlates with a proxy measure of cognitive reserve (years of education) in both healthy controls and individuals with Mild Cognitive Impairment (MCI), a clinical precursor of AD, and thus can be used as a potential biomarker of changes in cognitive reserve in patients at increased risk of AD. Also, the same group demonstrated that the global connectivity of the lateral frontal cortex correlates with greater education and slows down cognitive decline in patients with AD. Our study reveals that intraregional functional connectivity of LPFC positively correlates with IQ in healthy individuals. Such correlation was observed for all forms of the optical signal (HbO, HbR and HbT) showing the highest significance of the correlation with IQ for the total hemoglobin (HbT). Although we used individual IQ scores as a measure of cognitive reserve instead of the number of years of education, our results are in good agreement with the results of Franzmeier et al. (2017) and Franzmeier et al., 2018 [35,36]. Interestingly, the relationship between the PFC connectivity and the results of the Purdue Pegboard Test was quite different. Prefrontal connectivity did not correlate with the scores of the PP Assembly subtest and negatively correlated with the scores of the PP Both Hands subtest. These data can be understood and interpreted in the context of the functional role of the frontoparietal network. The FPN is the major component of the cognitive control network which is functionally distinct from other more specific networks including the sensorimotor network which is involved in motor functions. Thus, the lack of or negative correlation between frontal functional connectivity and motoric skills (as measured by PPT) can be expected. Along with the positive correlation with IQ, these data serve as further evidence for cognitive specificity of the frontoparietal network.

## 5. Conclusion

Our data provide further confirmation that cognitive reserve depends on the functional integrity of the lateral prefrontal cortex, a major cognitive control hub. Also, our experimental approach demonstrates that functional connectivity can be assessed by optical imaging using the fNIRS technology. As a cost-efficient and noninvasive technology, fNIRS can be used in the development of new neurophysiological measures of cognitive reserve with numerous possible applications in the context of healthy aging and cognitive disorders.

## Data Availability

The data used in this study are available on request from the author.
